# Faculty Insights on Artificial Intelligence Chatbot Integration in Dental Implant Clinical Education

**DOI:** 10.1002/jdd.13933

**Published:** 2025-05-08

**Authors:** Qiao Fang, Fatemah Solmaz Afshari, Judy Chia‐Chun Yuan, Cortino Sukotjo

**Affiliations:** ^1^ Department of Restorative Dentistry, College of Dentistry University of Illinois Chicago Chicago Illinois USA; ^2^ Department of Prosthodontics, School of Dental Medicine University of Pittsburgh Pittsburgh Pennsylvania USA

## 1 Problem

In the predoctoral dental clinical education at the University of Illinois Chicago, College of Dentistry (UIC‐COD), faculty encounter substantial workloads addressing frequent student inquiries pertaining to implant‐related workflow protocols and procedures. Timely access to accurate clinical information is crucial, yet traditional resources often lack immediacy and interactivity. Given the increasing popularity of artificial intelligence (AI) chatbots in education, exploring faculty perceptions of integrating such technology into predoctoral dental implant clinical education was essential to address potential benefits and identify concerns prior to broad implementation.

## 2 Solution

**FIGURE 1:**
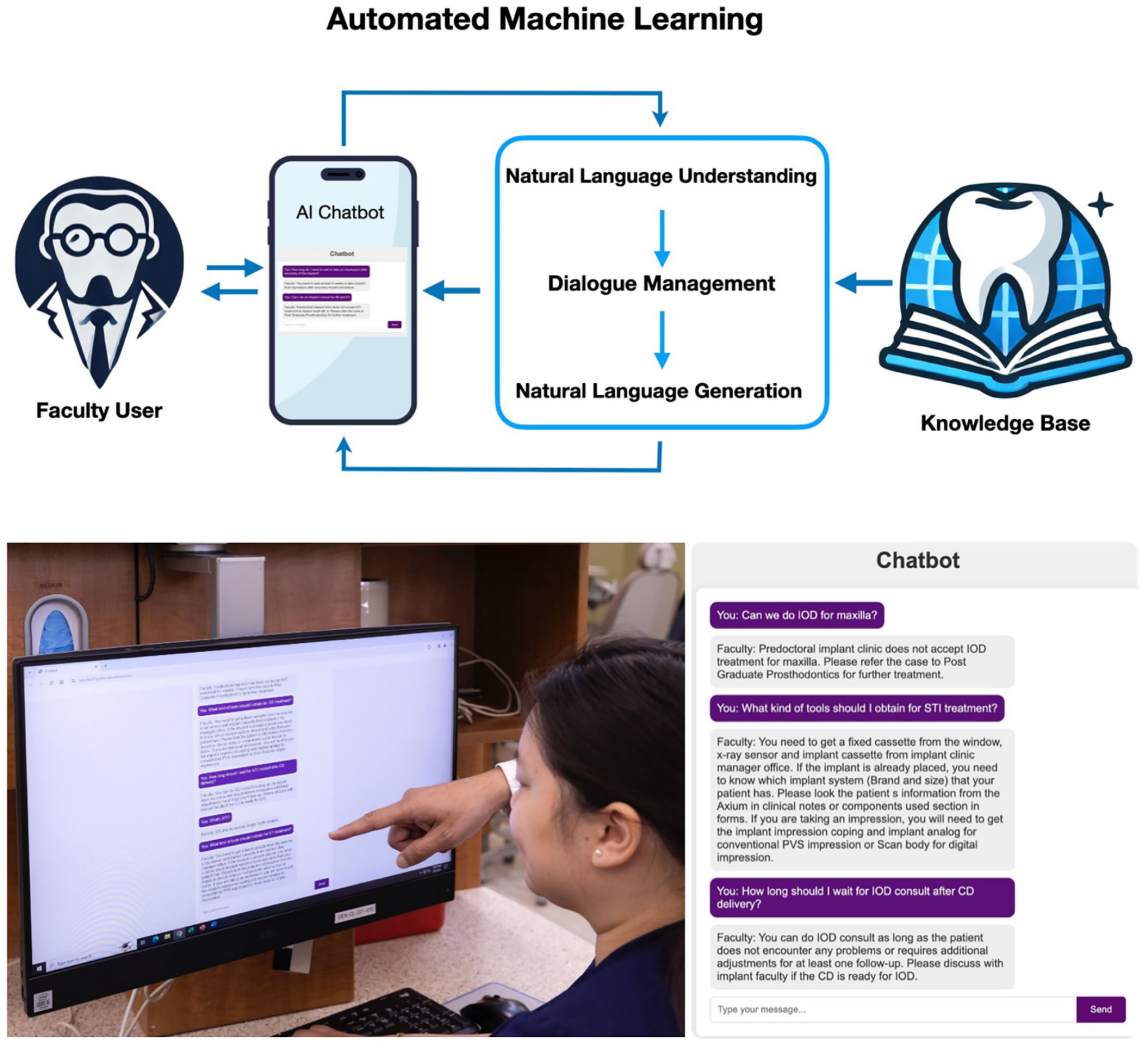
Architecture diagram and interface of Chatbot.

The authors piloted an AI Chatbot (Figure 1) specifically developed from a curated database of 1,300 implant‐related question‐answer pairs previously validated by predoctoral implant faculty at UIC‐COD. Twelve predoctoral faculty members from restorative and periodontics departments voluntarily consented to evaluate the Chatbot through clinical inquiry scenarios. Faculty interacted with the Chatbot and subsequently provided qualitative (advantages, disadvantages, and areas of improvement) via interviews and quantitative (structured Likert scale from strongly disagree = 1 to strongly agree = 5) feedback via a survey assessing AI awareness, user engagement, educational value, technological issues, and privacy and accuracy concerns.

## 3 Results

Participants reported strong adaptability to new technologies (mean ± SD; 4.1 ± 1.2) and recognized AI's potential to enhance educational experiences (4.5 ± 0.7). Faculty positively rated the Chatbot's timely responses (4.5 ± 0.5), its capability to reduce their clinical workload (4.4 ± 1.3), and its effectiveness in making learning more efficient (4.5 ± 0.9). The Chatbot was viewed as interactive (4.2 ± 1.1), user‐friendly (4.2 ± 0.7), and sufficiently valuable for faculty to recommend its use to peers (4.5 ± 2.0). Participants particularly emphasized the Chatbot's role in providing prompt, engaging, and anxiety‐reducing educational support. However, faculty concerns surfaced regarding Chatbot integration. Accuracy of provided information (3.9 ± 0.6), potential misleading responses (2.8 ± 1.3), privacy concerns (2.9 ± 1.0), questionable reliability of data sources (2.5 ± 0.8), and reduction of essential human interaction (1.9 ± 0.8) were identified as areas requiring careful attention (Table 1). Qualitative analysis (Table 2) indicated additional caution related to over‐reliance on Chatbot technology, the necessity for frequent updates, technical limitations, and the lengthiness of some responses. Faculty‐recommended areas for improvement include incorporating student feedback, integrating chatbots more seamlessly into the curriculum, providing multi‐language support, and enhancing data protection measures.

**TABLE 1:** Quantitative survey results.

Survey category	Survey questions	Chatbot (Mean ± SD)
**Awareness and Perception of AI**	I'm good at adapting to new technologies.	4.1 ± 1.2
	I've used Artificial Intelligence (AI) technology before.	4.1 ± 1.0
	I believe AI technology can make learning better.	4.5 ± 0.7
	I use educational AI resources regularly for academic inquiries.	2.6 ± 1.6
**Interaction with Chatbot**	I understand what Chatbot is and how it can be used in education.	4.3 ± 1.2
	Chatbots can be helpful and make learning more efficient.	4.5 ± 0.9
	The information provided by Chatbot is accurate.	3.9 ± 0.6
	The information provided by Chatbot is timely for my inquiries.	4.5 ± 0.5
	The depth and detail of Chatbot's information is sufficient.	3.4 ± 1.5
	Overall, I am satisfied with the quality of information provided by Chatbot.	4.2 ± 0.7
	Chatbot provides an interactive learning experience.	4.2 ± 1.1
	Chatbots can reduce the work burden of faculty in the clinic.	4.4 ± 1.3
	I'm willing to use Chatbot for learning.	4.7 ± 1.4
	Using Chatbot in the clinic can reduce my anxiety level.	3.6 ± 2.3
	I would recommend using Chatbot to my peers.	4.5 ± 2.0
**Concerns about using Chatbot**	Chatbots might provide incorrect or misleading information.	2.8 ± 1.3
	Chatbot's source of information is questionable.	2.5 ± 0.8
	Chatbots can replace human interaction.	1.9 ± 0.8
	I'm worried about privacy and security issues with using Chatbot.	2.9 ± 1.0

Likert Scale: I don't know (0), Strongly disagree (1), Disagree (2), Neutral (3), Agree (4), Strongly agree (5)

**TABLE 2:** Qualitative survey results.

Advantages	Disadvantages	Areas of improvement
Engaging learning experience	Over‐reliance on Chatbot versus independent learning	More descriptive terminology options (e.g., maxilla and upper jaw)
Customized learning paths	Potential for misleading answers	Incorporate student feedback & augmented reality
Reduced student anxiety	Limited interactivity & technical issues	Better integration with curriculum
24/7 accessibility	Responses can be too lengthy	Multi‐language support
Time efficiency for faculty	Requires frequent updates & maintenance	Stronger data protection measures

## 4 Lessons Learned

Faculty perceptions highlighted significant promise for the integration of AI chatbots into predoctoral dental implant clinical education. Faculty experienced a decrease in routine workload, students received timely and personalized assistance, and overall efficiency of clinical education efficiency improved. Nonetheless, ensuring content accuracy, preserving human interaction, and addressing privacy concerns remain critical priorities. Effective implementation of this innovation will require continuous content verification, rigorous training, maintenance of human oversight, and careful integration into existing educational frameworks.

The pilot study has shown Chatbot technology's potential to be a valuable adjunctive resource rather than a standalone replacement, indicating that careful, thoughtful adoption can advance dental education through innovation.

## Conflicts of Interest

The authors declare no conflicts of interest.
